# A rapidly time-varying equatorial jet in Jupiter’s deep interior

**DOI:** 10.1038/s41586-024-07046-3

**Published:** 2024-03-06

**Authors:** Jeremy Bloxham, Hao Cao, David J. Stevenson, John E. P. Connerney, Scott J. Bolton

**Affiliations:** 1 Department of Earth and Planetary Sciences, Harvard University, Cambridge, MA USA; 2 Department of Earth, Planetary and Space Sciences, University of California Los Angeles, Los Angeles, CA USA; 3 Division of Geological and Planetary Sciences, California Institute of Technology, Pasadena, CA USA; 4 Space Research Corporation, Annapolis, MD USA; 5 NASA Goddard Space Flight Center, Greenbelt, MD USA; 6 Southwest Research Institute, San Antonio, TX USA

**Keywords:** Giant planets, Core processes

## Abstract

Planetary magnetic fields provide a window into the otherwise largely inaccessible dynamics of a planet’s deep interior. In particular, interaction between fluid flow in electrically conducting interior regions and the magnetic field there gives rise to observable secular variation (time dependency) of the externally observed magnetic field. Secular variation of Jupiter’s field has recently been revealed^1–3^ and been shown to arise, in part, from an axisymmetric, equatorial jet^2^. Whether this jet is time dependent has not previously been addressed, yet it is of critical importance for understanding the dynamics of the planet’s interior. If steady, it would probably be a manifestation of deep dynamo convective flow (and jets are anticipated as part of that flow^4–9^) but if time dependent on a timescale much shorter than the convective turnover timescale of several hundred years, it would probably have a different origin. Here we show that the jet has a wavelike fluctuation with a period of roughly 4 years, strongly suggestive of the presence of a torsional oscillation^10^ (a cylindrically symmetric oscillating flow about the rotation axis) or a localized Alfvén wave in Jupiter’s metallic hydrogen interior. This opens a pathway towards revealing otherwise hidden aspects of the magnetic field within the metallic hydrogen region and hence constraining the dynamo that generates Jupiter’s magnetic field.

## Abstract (web summary)

An axisymmetric, equatorial jet in Jupiter’s interior has a wavelike fluctuation with a 4-year period, revealing hidden aspects of the magnetic field within the metallic hydrogen region and constraining the dynamo that generates the magnetic field.

## Main

**Fig. 1:**
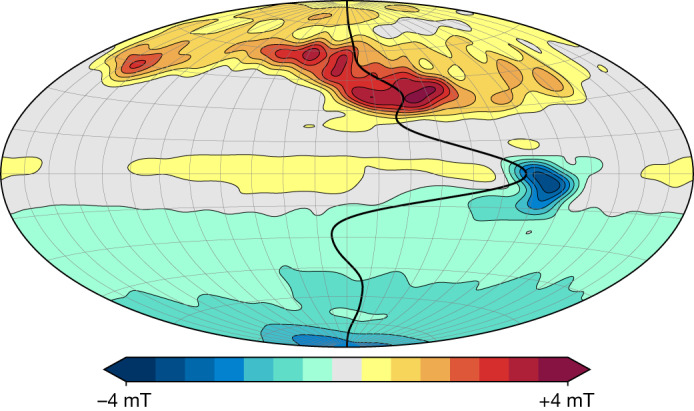
The steady velocity field and the background radial component of the magnetic field at 0.9 *R*_*J*_ . The projection is Hammer equal-area with the central meridian at 180° in System III coordinates (highlighted in grey); the central meridian is the zero line for the steady flow. The colour scale for the background magnetic field model is linear between the indicated limits. The flow velocity is scaled with latitude to account for the poleward convergence of meridians; the peak velocity (corresponding to the equatorial jet) is 0.86 cm s^−1^.

In Fig. 1, we superimpose a steady, axisymmetric, zonal flow profile on a background map of the magnetic field^2^ derived from Juno magnetic field observations^11^ from the spacecraft’s first 33 orbits. The flow is dominated by an equatorial jet, which induces intense secular variation in the vicinity of the Great Blue Spot (the region of concentrated field at the equator) as the magnetic field associated with this spot is swept eastwards. Owing to its dominant role in generating the secular variation^1–3,12^, a recent set of orbits by the Juno spacecraft^13^ was targeted at this region.

**Fig. 2:**
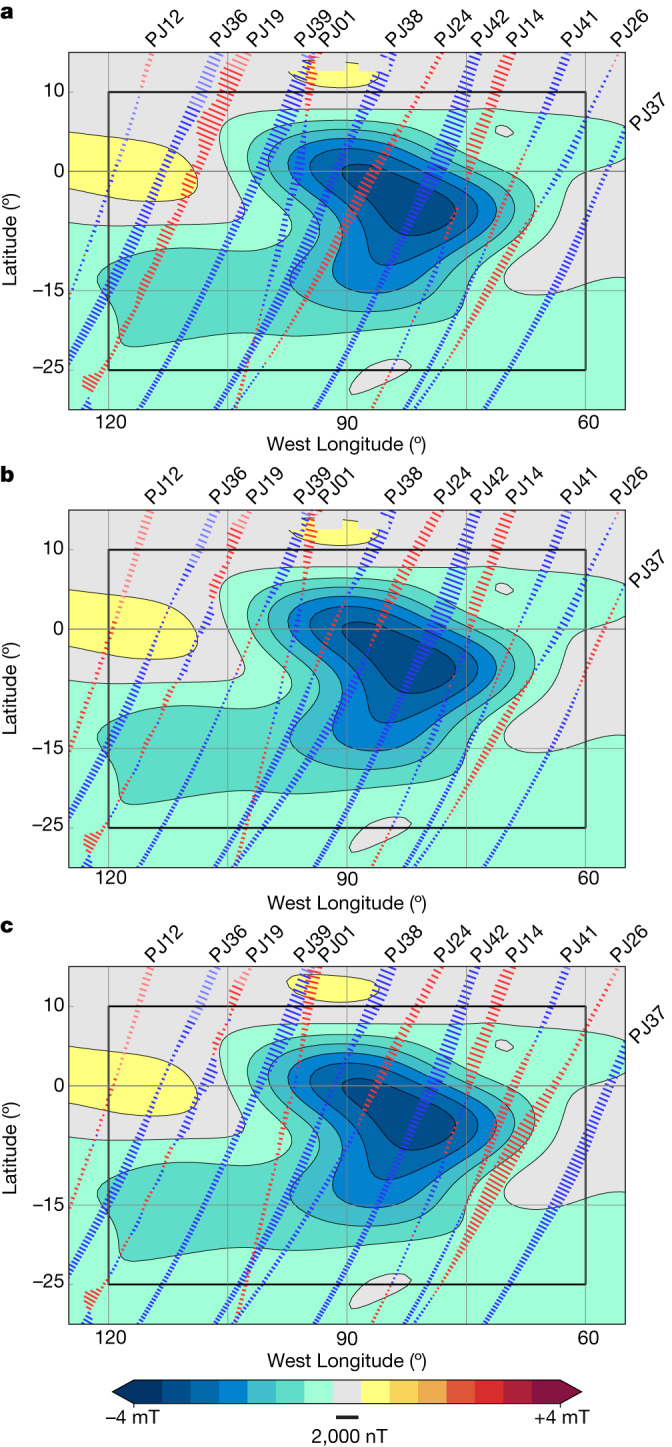
Residuals of the radial component of the magnetic field data along track. The residuals (the difference between the observation and the model prediction), calculated every 15 s, are plotted along the track, with positive residuals plotted west of the track (in red) and negative residuals east of the track (in blue) as the spacecraft passes through periapsis from north to south. The radial component of the magnetic field model is shown in the background. The projection is cylindrical with a grid spacing of 15°; the equator is highlighted in grey. The residuals are calculated within the box shown in black. The colour scale is linear between the indicated limits and the bar below the colour scale depicts the residual scale. **a**, The residuals from the 42-orbit steady flow model. **b**, The residuals from the 42-orbit steady flow model after applying the pass-by-pass velocity scale factors. **c**, The residuals from the 42-orbit steady flow model after applying the sinusoidal flow time-variation model.

To begin, we produce a new model including the magnetic field observations from these targeted passes (and other subsequent orbits over other regions of the planet). The model is produced using the same method as the model in Fig. 1 (Methods and ref. ^2^). One pass, PJ02 (in which PJ stands for perijove), did not acquire any data, so the number of data-yielding orbits is 41 compared with 32 orbits for the earlier model; note we refer to these models in terms of the last orbit used, that is, the 33-orbit model (Fig. 1) and the 42-orbit model. The resulting 42-orbit model has a global misfit of 492 nT compared with 411 nT for the 33-orbit model (for comparison, the root-mean-square (r.m.s.) field strength of the observations is 282,000 nT); within a box around the spot (Fig. 2) the misfit is 934 nT compared with 675 nT (where the r.m.s. field strength is 393,000 nT) and the maximum speed of the equatorial jet is 0.64 cm s^−1^ compared with 0.86 cm s^−1^. Thus, the fits we obtain to the 42-orbit dataset are poorer than those to the earlier 33-orbit dataset, especially near the spot, indicating that a steady flow performs worse as the time interval spanned by the passes increases. We may have expected, instead, that with the addition of these later passes that the misfit would decrease because these passes are at higher altitude over the spot and hence sample weaker field. Except for the southern hemisphere south of 30 °S, where the flow resolution is poor^2^, the flow profiles are broadly similar; however, the equatorial jet speed is reduced by 26% in the 42-orbit solution, suggesting that the flow may be changing with time. The pattern of residuals in Fig. 2a lends additional support to this possibility: we can identify pairs of passes that are spatially adjacent but separated in time that have oppositely signed residuals over the spot, notably PJ19 and PJ36, and PJ24 and PJ38. Oppositely signed residuals will result for adjacent passes if the actual flow speed at the time of the passes is greater than the steady flow solution for one pass and smaller for the other.

To examine the possibility that the flow speed is varying, we allow the flow to vary in amplitude on a pass-by-pass basis. We do this by applying a velocity scale factor to the flow for each pass (Methods). The velocity scale factor does not change the flow profile, instead it simply scales its amplitude. By doing so, we find the adjusted flow speed that gives the best fit for a particular pass, but for a different pass that flow speed will probably be different. These adjusted flow speeds represent the average flow speed from the baseline epoch of 2016.5 for each particular pass. In Fig. 2b we show the residuals after applying velocity scale factors to each pass. The residuals are reduced, especially for passes to the west of the spot. The misfit within the box is 721 nT, a variance reduction of 40% from the steady flow solution. This variance reduction can be considered as the maximum that can be achieved simply by varying the flow speed. However, this variation is only physically reasonable if we can find a time-varying flow consistent with the pass-by-pass velocity scale factors, in other words a time-varying flow that yields the corresponding average flow speed for each pass. It is possible, instead, that the different velocity scale factors (or average flow speeds) are mutually inconsistent.

**Fig. 3:**
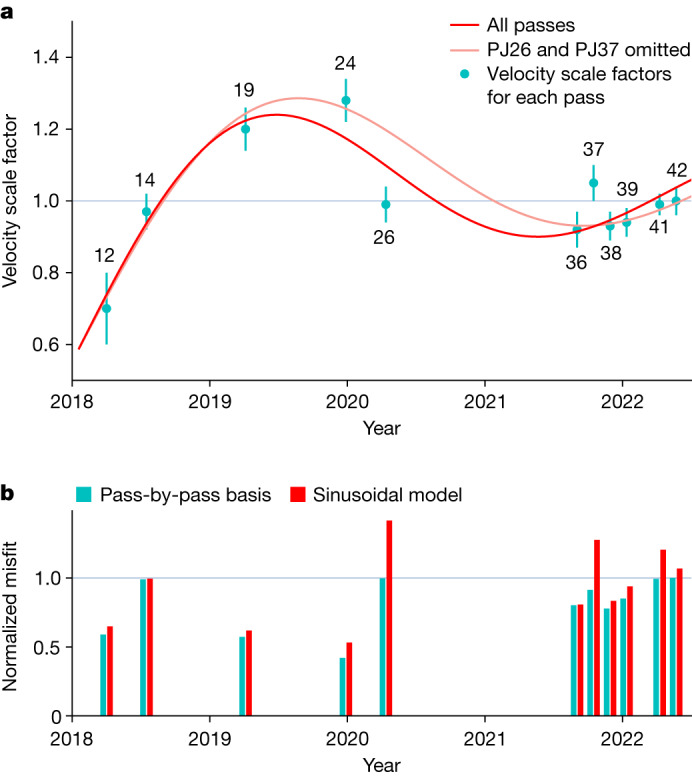
Velocity scale factors, time-averaged sinusoid fit and misfits. **a**, The cyan symbols represent the velocity scale factors for each pass. The error bars represent one standard deviation (Methods). The red curve shows the sinusoidal fit using all the passes and the light red curve the fit omitting PJ26 and PJ37 (for details of the fit, see Methods, equation (12). **b**, The misfit to each pass, normalized by the misfit to the 42-orbit steady flow model. The cyan bars represent the normalized misfit after applying velocity scale factors on a pass-by-pass basis; the red bars represent the normalized misfit after applying the sinusoidal model. In both panels, we depict the 42-orbit steady flow model by a horizontal line. **a**, The line corresponds to the unadjusted velocity of the 42-orbit steady flow model, in other words a velocity scale factor of unity for all passes. **b**, The horizontal line shows a misfit of 1, as the misfits have been normalized to the 42-orbit steady flow model. Source data

We examine whether such a flow exists by fitting the pass-by-pass velocity scale factors with a simple sinusoidally varying flow model with a single period and no damping (Methods). We omit PJ01 from this analysis as that orbit passes over the spot less than 2 months after the baseline epoch and thus is insensitive to variations in the flow (the flow would advect the spot by less than 0.05° during those 2 months). The best-fit solution is shown in Figs. 2c and 3: it has a period of 3.8 years and results in a variance reduction within the box of 24.8%. As expected, the variance reduction on a pass-by-pass basis varies substantially (Fig. 3b), as those passes with velocity scale factors that differ substantially from unity will have their fit enhanced more than a pass with a factor close to unity. Note that Fig. 3 shows the residuals to the radial component of the field, rather to the three components of the magnetic field, as the radial component is more readily interpreted in terms of changes in the flow speed. In a few cases, though, other components of the field show a much larger reduction in misfit than the radial component, most particularly *B*_*ϕ*_ (the east component of the magnetic field) for PJ24. In other words, there is not necessarily a one-to-one correspondence between the residuals in Fig. 2 and the variance reductions in Fig. 3. Comparing Fig. 2a with 2c, we can see that the residuals of the pairs of passes discussed earlier (PJ19 and 36, and 24 and 38) are much reduced. For most passes, the red bars in Fig. 3b (the normalized misfits to the sinusoidal model) are below the grey line corresponding to 1 (the normalized misfit of the 42-orbit steady flow model), but two passes (PJ26 and PJ37) stand well-above the grey line indicating that they are fit worse by the sinusoidal model than by the 42-orbit steady flow model. These two passes are the most easterly passes within the box. PJ37 requires a flow speed almost 15% more rapid than that of PJ36 and PJ38, which though temporally adjacent to PJ37 are not spatially adjacent to PJ37, indicating that additional spatial complexity in the flow may be required. PJ26 is, instead, fit by a slower flow than the sinusoidal model arguing instead for additional temporal complexity. Additional complexity could take the form of more than one wave being present or wave damping. In case our results are skewed by these two passes, we repeat the sinusoidal fit omitting them, as shown by the light red curve in Fig. 3a. The fit to most of the remaining passes, in particular PJ24 and the targeted passes (PJ36, PJ38, PJ39, PJ41 and PJ42) is improved. The period of the sinusoidal fit is changed by only a small amount from 3.8 to 4.1 years.

The period of roughly 4 years suggests that this is a torsional oscillation or Alfvén wave rather than, for example, a MAC (magnetic-Archimedean-Coriolis) wave^14^, which would have a much longer period. Torsional oscillations have also been proposed as the origin of cloud level variability in Jupiter on subdecadal timescales^15^: the zonal shear associated with a torsional oscillation may modulate the heat flux from the deep interior, which may in turn result in variability of observed infrared emissions at cloud level. The wave speed of torsional oscillations is determined by the r.m.s. value of the component of the magnetic field, ${\bar{B}}_{{\rm{s}}}$, perpendicular to the rotation axis^10^ (where the average is taken over longitude and the latitude band of interest). For an equatorial belt of ±10° about the equator (the latitudinal extent of the deep equatorial jet), we find ${\bar{B}}_{{\rm{s}}}=0.6$ mT at 0.9 *R*_*J*_. This corresponds to an Alfvén wave speed of 10^−2^ ms^−1^.

The period of the oscillation depends, of course, on its wavenumber *k*, for which we have no direct observation. If the cloud level variability is due to torsional oscillations, then the wavenumber can be estimated from the length scale of those variations, yielding dimensionless wavenumbers *kR*_J_/2π in the range 10 to 15 (ref. ^15^). Here, however, we are examining a single equatorial fluctuation rather than a set of torsional oscillations spanning a wide range of latitudes. For the equatorial jet, a dimensionless wavenumber of 10 could be considered (although this would be based on the azimuthal extent of the jet rather than its wavenumber in the *s* direction) yielding a period of roughly 15 years: that is, four times longer than that found here.

However, our estimate of ${\bar{B}}_{{\rm{s}}}$ may be too small: the field is most probably stronger at depths below 0.9 *R*_*J*_, but the field below that depth cannot be reliably estimated from the externally observed potential field owing to the rapid increase of electrical conductivity with depth^16^; and second, intense, small scale magnetic fields (which will be geometrically attenuated in the observations at satellite altitude) may serve to increase ${\bar{B}}_{{\rm{s}}}$ further.

A period of 4 years corresponds to a field strength ${\bar{B}}_{{\rm{s}}}\approx 3$ mT, similar to the field strength associated with the spot itself, so the wave may instead be a localized Alfvén wave propagating along the field lines associated with the spot (which are largely in the *s* direction), rather than an axisymmetric torsional oscillation, in which case a superimposed longer period torsional oscillation may then also be excited.

## Methods

### Model equations

We write the radial component of the magnetic induction equation^17^ in the form^2^

1 $$\frac{\partial {B}_{r}}{\partial t}=-\frac{1}{r\sin \theta }{w}_{\phi }(\theta )\frac{\partial {B}_{r}}{\partial \phi }(\theta ,\phi )$$

where *w*_*ϕ*_(*θ*) is the zonal flow velocity (assumed steady in time), and (*r*, *θ*, *ϕ*) are spherical polar coordinates.

We expand the magnetic field using a standard spherical harmonic expansion^18^ with coefficients **g**, and the zonal flow velocity as a Legendre expansion

2 $${w}_{\phi }=-\sum _{{\ell }}{v}_{{\ell }}\frac{{\rm{d}}{P}_{{\ell }}^{0}}{{\rm{d}}\theta }$$

with coefficients **v**_0_ = {*v*_*ℓ*_}. We integrate in time from an initial time *t*_0_, giving

3 $${\bf{g}}(\tau )={\bf{g}}(0)+({\int }_{0}^{\tau }G(\,{\bf{g}})\,{\rm{d}}\tau )\,{{\bf{v}}}_{0}$$

or equivalently

4 $${\bf{g}}(\tau )={\bf{g}}(0)+V({{\bf{v}}}_{0}){\int }_{0}^{\tau }\,{\bf{g}}\,{\rm{d}}\tau $$

where *τ* = *t* − *t*_0_. The elements of the matrices *G*(**g**) and *V*(**v**_0_) depend on Elsasser integrals^19^.

If the data are represented by a vector **y**, then we can write

5 $${\bf{y}}={\bf{f}}({\bf{m}})+{\bf{e}}$$

where **m** = {**g**(0) : **v**_0_} and **e** represent the data errors.

Solutions to the nonlinear inverse problem, at a reference radius of *r* = 0.9 *R*_*J*_, are found iteratively using a regularized inversion^2^.

### Residuals and misfit

The residual vector **r** is defined by

6 $${\bf{r}}={\bf{y}}-{\bf{f}}({\bf{m}})$$

and the misfit by

7 $$\sqrt{{{\bf{r}}}^{T}{\bf{r}}/N}$$

where *N* is the length of **y**, that is, the number of data.

### Velocity scaling factors

We scale the velocity coefficients with a simple scalar factor for each pass

8 $${v}^{{f}_{i}}={f}_{i}\,{v}_{0}$$

where the subscript *i* refers to the perijove *i*. Note that when the scale factor *f*_*i*_ is applied to pass *i*, the resulting scaled velocity is used over the entire time interval 0 to *τ*_*i*_ when calculating the fit to pass *i*, in other words the scaled velocity is the best-fit average velocity over the time interval for each particular pass.

Owing to the presence of correlated errors in the data and the effects of unmodelled signals, calculating error bars on the velocity scaling factors is itself highly uncertain. We adopt the following procedure: given the typical field intensity measured by Juno over the box around the spot is roughly 500,000 nT, and the nominal uncertainty is 0.01% (ref. ^11^), we examine the range of scaling factors that give r.m.s. misfits within 50 nT of the minimum to estimate the errors.

### Fitting velocity scaling factors with a periodically varying flow

We wish to test whether augmenting the steady flow with a periodic component can explain the velocity scaling factors on a pass-by-pass basis. We model a periodic component with a simple sinusoid

9 $$v(\tau )=\left(C+A\cos \left(\frac{2{\rm{\pi }}\tau }{T}+\psi \right)\right){v}_{0}$$

and seek to relate the velocity scaling factors {*f*_*i*_} to the scale *C*, amplitude *A*, period *T* and phase *ψ*. This equation gives the instantaneous velocity at each time *τ*, but the velocity scale factors measure the average velocity over the time interval from *τ* = 0 to *τ*_*i*_.

Thus, for the perijove at time *τ*_*i*_ the scale factor *f*_*i*_ is given by the average velocity over the time interval *τ* = 0 to *τ*_*i*_

10 $${f}_{i}=C+\frac{A}{{\tau }_{i}}{\int }_{0}^{{\tau }_{i}}\cos \left(\frac{2{\rm{\pi }}\tau }{T}+\psi \right){\rm{d}}\tau $$

11 $$=\,C+\frac{A}{{\tau }_{i}}\frac{T}{2{\rm{\pi }}}\left[\sin (\frac{2{\rm{\pi }}{\tau }_{i}}{T}+\psi )-\sin (\psi )\right]$$

We use the nine included orbits to estimate *C*, *A*, *T* and *ψ* using a simple search method. We weight all the orbits equally so as not to unduly bias the fit towards the later orbits that are closely clustered in time and which have smaller errors (Fig. 3a). The best-fit solution has [*C*, *A*, *T*, *ψ*] = [1.08, 1.13, 3.83 years, 2.82 rad]. The fit is shown in Fig. 3.

Then

12 $${\bf{g}}({\tau }_{i})={\bf{g}}(0)+{\int }_{0}^{{\tau }_{i}}V(({\bf{v}}(\tau )){\bf{g}}(\tau )\,{\rm{d}}\tau $$

from which we can compute the misfit to the observations.

## Online content

Any methods, additional references, Nature Portfolio reporting summaries, source data, extended data, supplementary information, acknowledgements, peer review information; details of author contributions and competing interests; and statements of data and code availability are available at 10.1038/s41586-024-07046-3.

### Source data

Source Data Fig. 3

## Data Availability

All data used in this study are available from the NASA Planetary Data System (https://pds.nasa.gov). The 42-orbit model can be downloaded from 10.7910/DVN/HFFI7A. Source data are provided with this paper.

## Code availability

Example code to read the 42-orbit model can be downloaded from 10.7910/DVN/HFFI7A.
